# Knowledge-based analysis of proteomics data

**DOI:** 10.1186/1471-2105-13-S16-S13

**Published:** 2012-11-05

**Authors:** Marina Bessarabova, Alexander Ishkin, Lellean JeBailey, Tatiana Nikolskaya, Yuri Nikolsky

**Affiliations:** 1 Thomson Reuters, IP & Science, 5901 Priestly Dr., #200, Carlsbad, CA 92008, USA

## Introduction

The analytical methods in proteomics can be roughly divided onto statistical and "functional" approaches. Some statistical methods are applied for identification and quantization of proteins in complex individual peptide profiles. Another set of tools (machine learning and classification algorithms [1]) helps to evaluate performance of selected proteins as descriptors in multi-sample studies, for instance as prognostic biomarkers in disease or drug response diagnostics. Technically, neither of these applications requires any knowledge of the protein relevance to disease pathology. Statistical analysis alone could be sufficient for most clinical applications of proteomics-derived biomarkers - provided their high enough performance on large populations (accuracy, sensitivity and specificity).

However, human (and mammalian in general) biology is too complex to be handled by statistical approaches alone. For one (just it is the case with other OMICs technologies like global gene expression or SNP genotyping), proteomics profiles in different samples are highly heterogeneous for the same clinical phenotype (e.g., disease survival rate or drug response). The best performing clinical protein biomarker, PSA, is elevated in less than 50% of prostate cancer patients, and variability of individual proteins in multi-variant proteomics profiles is usually substantially higher. Secondly, protein profiles derived either from bodily fluids or solid tissues biopsies are not sufficiently selective for most phenotypic endpoints and its statistical association with different diseases and drug responses can be misleading. For example, almost any of >5,000 human diseases is associated with inflammation. However, statistically processed proteomics profiles usually do not allow to distinguish between them. In order to deconvolute these complexities, one needs tools and databases for functional, or "pathway", analysis. Functional analysis utilizes accumulated knowledge about relationships between proteins in a living cell to interpret the experimental data instead of relying on data only.

There are two important technical aspects for biological interpretation of proteomics data. First, proteomics is "high-throughput", or OMICs, technology, meaning that the outcome of proteomics experiments is a *list *of proteins differentially modified or abundant in a certain phenotype. The mere size of proteomics datasets requires specialized analytical tools, which deal with large *lists *of objects, rather than individual proteins, one at a time. Second, proteomics profiles are usually "global" in terms of sample source, i.e. they represent snapshots of a whole blood profile or tissue biopsies, which defines a very complex temporal, cell type- and tissue-specific biological context for protein activity. On average, a human protein has over 20 physical interactions of different types with other proteins, nucleic acids and metabolites and participates in dozens of biological pathways and processes (MetaCore database, Thomson Reuters). Furthermore, alterations in the same protein/gene (mutations, epigenetic changes, RNA splice variants, phosphorylated proteins, isoforms etc.) can be associated with dozens of diseases and conditions. In most cases, the mechanisms of such associations are unknown. On the other hand, there are a huge number of facts and findings about different aspects of functionality of these proteins in different tissues, cell lines and conditions, scattered in hundreds of thousands of experimental articles. What is the value and relevance of this "accumulated knowledge" for the analysis of an individual proteomic profile and how could it be applied in meaningful way?

The first step is to assemble all relevant published data in a computer-readable form, then index and structure this content to make it accessible for automated search and analysis applications. Over the last decade, a variety of text mining algorithms and manual annotation techniques have been developed to extract primary and meta-data from experimental literature, patents and other written sources (MedScan by Ariadne Genomics [2]; I2E by Linguamatics [3]). In addition, several large-scale annotation or editorial projects in the public domain have been initiated, monitored and completed by the industry, and have lead to comprehensive "knowledge" databases. These knowledge bases include MetaCore (Thomson Reuters), IPA (Ingenuity), KEGG [4] and HPRD [5], to name a few. The main types of data stored in these databases deal with protein functionality represented as physical and functional protein interactions of different types (often assembled into multi-step pathways) and gene/protein - phenotype associations linking genes and protein variants to the diseases, toxic effects, drug responses and other "end points". Manually curated "knowledge" databases have rich semantics in a form of functional ontologies and controlled vocabularies of terms and synonyms. Genes, proteins, metabolic compounds and drugs are assigned to different entities, or terms, in multiple ontologies, for instance cellular processes or standardized protein functions as it is done by GeneOntology consortium [6]. Sub-categorization of proteins and genes into ontologies and representation of protein functionality as binary interactions (please see "network analysis tools" section for a definition on an interaction) and multi-step pathways are the two pre-requisites needed for applying tools of functional, or "knowledge-based" analysis.

Functional analysis of proteomics data can be divided into two types, dealing with proteins as objects and protein interactions, correspondingly. The first one, known as "ontology enrichment analysis" shows how different ontology terms (pathways, processes, disease biomarkers etc.) are relatively represented in the proteomics profiles (i.e. lists, or sets of proteins revealed by proteomics experiments) [7]. The second type of analysis evaluates protein's functionality represented as silos of its interactions with the proteins on the list of interest. The core assumption is that relative connectivity of a protein reflects its functional importance for the phenotype [8]. Relative connectivity can be calculated as a number of interactions between the given protein with the proteins on the list of interest normalized to the number of interactions it has with all proteins. Relative connectivity with a given protein list of interest can be calculated for every protein in the organism's "proteome" (human proteome is defined as about 24,000 proteins with experimentally determined function) (*interactome *tools) and for the subsets of proteins and interactions represented as *networks*.

Here, we present the main statistical tools for enrichment and interactions-based network analysis applied in the MetaCore/MetaDrug analysis platform (Thomson Reuters) with demonstrated examples of proteomics studies analyzed with the system.

## Enrichment analysis in functional ontologies

### Principle

Ontology enrichment is the most ubiquitous type of functional analysis, which evaluates relative representation of biological functions, or ontology terms, such as pathways and cell processes, for the proteomics profile of interest. Enrichment analysis (EA) consists of "mapping" (matching identifiers) of experimental data (proteomics list or profiles) onto terms of functional ontologies (pathways, disease biomarkers etc.) followed by ranking the resulting ontology terms based on the size of the identifier's (ID) intersection between the term and the experimental data. "Enrichment" is thereby calculated as a probability of the observed overlap between the genes/proteins from the experiment and the selected ontology term. There are two main types of enrichment analysis algorithms. One, a "whole set" approach, ranks proteins by evidence of differential abundance only (without a decision of abundance cut-offs). Another set of algorithms requires a pre-calculated set of proteins, usually selected by abundance fold change and statistical significance thresholds. The former "whole set" approach is implemented in Gene Set Enrichment Analysis (GSEA) [9] or Parametric Analysis of Gene Set Enrichment (PAGE) [10] algorithms. Gene set approach is realized in several algorithms, such as hypergeometric test [11]. In the whole data set approaches, the pathways and other ontology terms are ranked according to their association with the protein or gene expression changes between two sample groups for *every *protein/gene in the set. On the contrary, gene (protein) list based algorithms work only with a subset of proteins and, therefore, require pre-selection of significance threshold for expression change at the protein level. The algorithms then use only information about protein content of the list, regardless of protein expression values. A list of differentially abundant proteins identified by t-test between two groups of samples in the whole data set is an example of input data in this approach.

The hypergeometric test evaluates significance of an association between the two kinds of categorical classification for a set of objects (for example, presence of a protein in the list of interest and its belonging to a pathway or any other ontology term). In the case of enrichment analysis, the intersection between a protein list of interest and a list of proteins involved in a certain pathway is calculated. Under the null hypothesis of no association, the probability of occurrence of an intersection of a given size by chance follows the hypergeometric distribution.

### Calculation of an enrichment distribution of ontology terms

**Figure 1:**
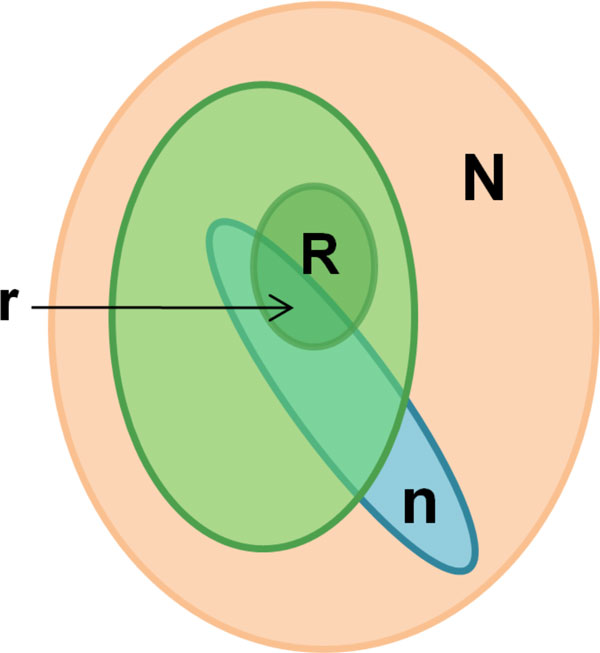
**P-value based ranking in ontology enrichment analysis**. Subset ***N ***represents a complete human proteome (all proteins and complexes in MetaCore database). The subset ***n ***of these nodes corresponds to the experiment. Light green ellipse depicts the set of ontology categories, which can be hierarchically ordered, (as is the case for GO processes or MeSH disease classification). R-set is the union of all the proteins tied to the particular characteristic or category (e.g., proteins associated with at least one GO processes).

Let us consider a set of size *N*, representing all nodes (proteins and complexes) in MetaCore database, which we consider as the "universe" (Figure 1). The "marked" subset *R *of the universe *N *defines the proteins of interest. There are two notable points: 1) generally not all the proteins from the list of interest can be associated with nodes in the network of protein interactions; 2) some proteins may correspond to multiple nodes and some nodes may correspond to multiple proteins (i.e. protein complexes). n is another subset *N *which defined functionally determined set of nodes Subset n can be either pre-established (known pathway, cellular processes or disease biomarkers) or built "on the fly" using a content of a protein interaction database.

There may be some marked nodes among the *n *nodes of the module. The probability of a subset of size "*n" *to include *"r" *marked ones provided that n and *R *are unrelated follows the hypergeometric distribution:

$$p\left(r,n,R,N\right)=\frac{\left(\begin{array}{c} R\\ r \end{array}\right)\left(\begin{array}{c} N-R\\ n-r \end{array}\right)}{\left(\begin{array}{c} N\\ n \end{array}\right)}$$

It is essential that these equations are invariant in terms of exchange of *n *for *R, which *means that the "subset" and "marked" are equivalent and symmetrical sets.

The null hypothesis of the enrichment test is that the node list of interest is not associated with an ontology term. The p-value of the test is calculated as cumulative probability of observing *'r' *or more nodes in the intersection under the null hypothesis:

$$pVal\left(r,n,R,N\right)=\overset{\text{min}\left(n,R\right)}{\underset{i=max\left(r,\;R+n-N\right)}{\sum }}p\left(i,n,R,N\right)$$

If the p-value is sufficiently small (conventionally, less than 0.05), the null hypothesis is rejected and the ontology term is called significantly enriched with the proteins of interest. The test is repeated for all terms in a given ontology, and all significant terms are returned, ordered by p-value of enrichment.

As ontologies typically contain many terms, some of them may turn out to be significant for particular list of interest and given p-value threshold just by chance. Thus, proper false discovery rate (FDR) control of enrichment analysis findings is required. In MetaCore, the FDR is controlled using Benjamini & Hochberg approach [12], which ensures that no more than 5% of significant terms are false positives.

### Selection of the background list for enrichment analysis

The null hypothesis of the hypergeometric test can be viewed as 'competitive' null hypothesis [7]. Comparing the intersection of those assesses the association of an ontology term with the node list of interest and the expected intersection of the same list with random node sets sampled from the same background 'universe' of nodes. Therefore, the test result strongly depends on the choice of the 'universe'.

Selection of an appropriate background 'universe' is often challenging in the high-throughput studies and may cause misleading results. The most conservative approach is to define the 'universe' as the complete set of genes or proteins measured by a particular high-throughput assay. For example, a subset of genes differentially expressed in breast cancer has to be tested for enrichment using the gene content of the microarray it was generated on, not the whole set of human genes. In proteomics, the background list can be defined as a complete set of proteins known to be expressed in an organ/tissue/body liquid/cell line of sample origin. This is important, as only a fraction (about 10%) of human protein-encoding genes are noticeably expressed in any given tissue [13], and only a subset of the expressed genes can be detected in a proteomics experiment. In MetaCore, the gene content of commercial microarrays, custom gene/protein sets, species and orthologs, cell processes and other functional groups can be selected as the background lists.

The background list is limited by the protein content of the ontology applied for enrichment; for instance, a non-redundant union of all human "canonical" pathways. Proper selection of the ontology is very important, as the enrichment p-values vary depending on the size of the examined protein list and the selection of the background. The most complete ontology of human canonical pathways (1200 pathway maps in MetaCore) has about 9,000 proteins, as compared with >24,000 human proteins with experimentally determined function which have at least one interaction each.

The ontology term "*n*" can represent a number of proteins selected based on some common property, for instance, belonging to the same ontology term and sharing the same annotation. Therefore, enrichment is only as informative as the ontology behind it. Analysis with only one ontology (e.g., GO processes) provides a "one dimensional" overview of a dataset. Ideally, the ontologies should be specifically designed for an application. A toxicoproteomics dataset should be evaluated against ontology of organ-specific histopathology and prostate cancer datasets against an annotated ontology of prostate cancer biomarkers and pathology pathways.

### Ontologies for enrichment analysis

On average, human proteins have over 20 direct interactions and can participate in dozens of pathways, cellular processes and complexes, depending on context. Moreover, several "hub" proteins such as p53 and NF-kB, are much more ubiquitous and have over 1,000 interactions. In order to deal with such complexity, each protein has to be well functionally annotated, i.e. its function assigned to certain ontology terms by experimental evidence. This can be achieved by expert manual curation of full text experimental literature, a time consuming and tedious process. There are hundreds of biologically relevant ontologies available, although only some of them are sufficiently populated with proteins for enrichment analysis. Arguably, the best-known public ontologies are the ones developed by Gene Ontology (GO) consortium [6] for cellular processes, protein functions and cellular localizations. KEGG [4] is a popular public ontology and database of metabolic and signalling pathways from multiple organisms including human. In general, ontologies are not well standardized, they often apply poorly overlapping ID systems. Currently, an industry-academy incentive known as Pistoia consortium, intends to unify and standardize public ontologies [14]. Commercial functional analysis platforms typically use a combination of publically available and proprietary ontologies and run enrichment analysis in each separately, one at a time. Below, we summarize the functional ontologies featured in the MetaCore database, where each ontology corresponds to a certain "dimension" of biological functionality.

• Signaling pathways. Multi-step chains of consecutive signaling interactions, typically consisting of a ligand-receptor interaction, an intra-cellular signal transduction cascade between receptor (R) and transcription factor (TF) and, finally, TF - target gene interaction. Signaling pathways are mainly used by network generation tools and for enrichment by direct access to the database.

• Metabolic pathways. Multi-step chains of metabolic reactions, linked into functionally self-sufficient linear chains and cycles. Fragments of metabolic pathways are shown as static images reachable from the protein pages. Metabolic pathways are also used for network generation and visualized on the networks.

• Canonical pathways maps. Pathway maps, or wire diagrams, is the most popular ontology for enrichment and the main type of pathway visualization in MetaCore. Pathway maps are interactive images drawn in a proprietary Java-based editor and typically contain 3-6 pathways. There are over 1200 pathway maps in MetaCore, comprehensively covering human signaling and metabolism, selected diseases and some drug targets mechanisms.

• Canonical pathway maps folders. All canonical maps are assembled into a hierarchical tree folder structure. The folders typically correspond to higher-level processes, such as "apoptosis", "cell cycle", or "amino acid metabolism". The folder structure can be visualized in a Browser mode and from enrichment analysis distributions.

• Cell process network models. This ontology represents Thomson Reuters' reconstruction of main signaling and metabolic processes in the cell, such as a "cell cycle checkpoints" or "innate immune response". The manually built process networks typically have over 100 nodes (proteins) belonging to a certain normal cellular processes. The edges are selected from MetaCore content.

• GO processes. These are a GUI-supported representation of the Gene Ontology (GO) collection of cellular processes, which is supported by GO tree structure and access to proteins and interactions within a process. This ontology is updated with GO standard updates. GO processes are mostly used in enrichment analysis and for prioritization of genes on the built networks.

• GO molecular functions. A GUI-supported ontology of standard protein functions from GO. Mostly used in enrichment analysis.

• Disease biomarkers. These are a collection of genes genetically linked to over 500 diseases and conditions, supported by the hierarchical disease tree and GUI for gene retrieval. Disease biomarkers are mostly used in enrichment analysis.

• GeneGo disease network models. GeneGo reconstruction of disease mechanisms in a form of manually built networks. These are mechanistic networks linking the disease-associated genes via physical and functional protein interactions.

• GeneGo toxicity networks. GeneGo reconstruction of toxicity mechanisms in a form of manually built networks. These are mechanistic networks linking genes associated with a particular toxicity endpoint via physical and functional protein interactions.

### Examples of ontology enrichment in proteomics studies

**Figure 2:**
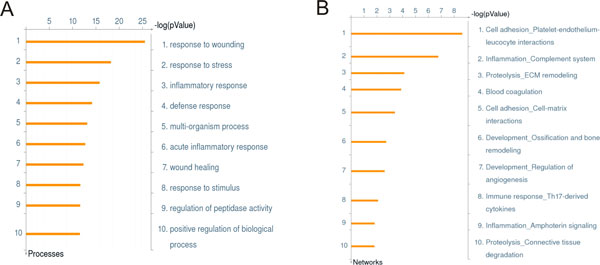
**Enrichment analysis of plasma proteome from an ovarian cancer mouse model**. A. Top 10 significant biological processes from GO ontology. Bar length reflects the significance and equals to the negative logarithm of enrichment p-value. B. Top 10 significant maps from MetaCore canonical pathway map ontology.

An example of ontology enrichment of proteomics data was demonstrated by Pitterri et al. [15]. Authors identified a subset of secreted proteins from a K-Ras/Pten ovarian cancer mouse model, using quantitative high-resolution mass spectrometry and shotgun LC-MS/MS analysis to identify potential early ovarian cancer biomarkers. A total of 58 plasma proteins with altered expression level during tumour development were subjected to enrichment analysis using MetaCore to determine their involvement in a functional, pathways context (Figure 2). The most prevalent processes across GeneGo ontologies and Gene Ontology included cell adhesion, proliferation, development and extracellular matrix remodelling as significant represented processes. This example demonstrates two valuable messages. The first is the ability to carry out quantitative analysis of the functions (in this example, canonical pathways) of the secreted serum proteins. The results are listed according to the -log p-value determined by the hypergeometric calculation, allowing an analyst to not only assess the represented functions but also determine which functions are more represented than others. The distribution of pathways or the top scored pathway can be applied as phenotype descriptors, for instance for patient stratification of disease sub-clustering (reviewed in [16]). The second point is the use of multiple functional ontologies (pathways, cell processes, disease biomarkers etc.) provides different viewpoints of functional representation of the proteomics set, based on the context of the ontology. In this example, both GO ( Figure 2, A) and GeneGo's proprietary GeneGo Process Network ontology ( Figure 2, B) support the observation of the role of inflammation in early ovarian tumorigenesis. However, the more descriptive GeneGo Process Network Ontology further delineates specific cell function in the inflammatory process to include extracellular matrix remodelling.

## Protein interaction - based analysis

Ontology enrichment is a "low resolution" analysis, which is useful for an overall description of functionality in the proteomic dataset and for functional focusing of the dataset by exporting proteins hitting a certain pathway or a process. However, relative distribution of pathways or other ontology terms cannot directly pinpoint the most important proteins on the pathway or in the dataset, i.e. ontology enrichment is not a self-sufficient hypothesis-generation tool. Ontology enrichment cannot rank individual proteins based on significance and answer questions such as "what is the most important protein kinase for my dataset?" or "what is the 'master switch' transcription factor to be knocked out in an animal model?" In order to answer such questions, one needs to consider the "local interactome" for each protein in the dataset and compare it with the global human interactome. The main assumption behind "knowledge-based" analysis is that it is the set of interactions, which defines protein functionality. One can identify the set of interactions between all 24,000 human proteins of known function with all the proteins in the dataset of interest and evaluate relative enrichment in interactions space for each human protein. The proteins which are statistically significantly "overconnected" with the given proteomics profile can be considered as the most important. Interactions enrichment can be carried out by two sets of tools, i.e. interactome and network analysis. The main difference between the two is that interactome methods calculate general enrichment of binary interactions around each object, and network methods apply rules (network building algorithms) for connecting binary interactions into multi-step modules.

It is important to note that the term "interaction" defined here is not restricted to physical binding between proteins, but include any relationships between proteins (and other objects) described by the following mechanisms: binding, cleavage, covalent modifications, phosphorylation, dephosphorylation, transformation, transport, catalysis, transcriptional regulation and microRNA binging. An "object" then can be any type of nucleic acid (mRNA, miRNA, DNA), gene, protein (with separate objects for a fusion protein or protein complex), compound or reaction (metabolic or transport). Each network object with at least one interaction is manually curated and the interactions retrieved from full text peer-reviewed literature. Each interaction is annotated with the following attributes:

- A causative effect for the interaction (positive, negative)

- A mechanism associated with this effect (binding, catalysis, transcriptional regulation etc.

- Direction

- Species (human, mouse or rat)

For each network objects, the information is derived from high quality experimental data. The collection of network objects and their relationships (edges) are provided in the underlying database.

### Interactome analysis

In MetaCore, "interactome" tools evaluate relative connectivity of each protein in a dataset of interest with every other human protein (within dataset or entire "global" human interactome). Since proteins work in groups (complexes and pathways), which are defined by interactions, it is assumed that relative connectivity reflects relevance, or importance, of a protein for a given dataset. For example, if a transcription factor has significantly more interactions than expected by chance with its targets in a proteomics profile from a primary prostate tumour, it is likely to be an important "master regulator" of cancerogenesis in this particular tumour. Identification of the whole set of over-connected proteins can help to reconstruct the biological mechanism the proteomics profile. The interactome methods are well suited for deducing signalling and regulation proteins which activation would lead to a phenotype that are connected to the proteomics dataset but undetectable by proteomics profiling. Proteomics datasets are often enriched in "effector" proteins, such as metabolic enzymes or structure proteins which encoding genes are constitutively expressed in a given sample and presented in higher abundance. Such functional bias is evident when proteomics profiles are compared with gene expression profiles from the same sample. On the contrary, regulatory genes are usually expressed transiently; many signalling proteins tend to degrade fast and are regulated on post-translational level leaving this subset of proteins undetectable. At the same time, signalling pathways are quite important as the source of conditional "triggers" (for instance, GPCRs, signal transduction protein kinases) and are often prime candidates for drug target development. Two "topology" methods described below help to deduce proteins, which are usually not present in proteomics datasets but closely connected with them by protein-protein interactions of different mechanisms.

#### Calculation of protein connectivity in interactome analysis

Connectivity between proteins is carried out as follows: At least 2 protein populations are considered: 1) the proteins in the uploaded dataset (i.e. proteomics list) of interest (local interactome) and 2) the proteins in a background list (global interactome). The algorithm calculates the relative connectivity between the local interactome and compares it to the general interactome. First, one-step (interaction) neighbours are identified around individual proteins. Then, the procedure calculates the main properties of the "local" interactome for the proteomics dataset of interest. The "local" interactome is defined either as the compilation of all interactions between the genes/proteins within the uploaded list/experiment or as a set of all direct network neighbours of the proteins from the uploaded list and interactions between them. The interactome properties include:

*Degree *, the average number of protein interactions per protein from a given set [17]. Since the interactions are directed, the nodes can be characterized by IN and OUT-degree, i.e. the average number of outgoing and incoming interactions.

*Clustering coefficient*. This captures the density of connectivity between the protein's neighbours [17]. It is defined as: $C_{i}=\frac{2n_{i}}{k_{i}\left(k_{i}-1\right)}$, where *Ni *is the number of interactions between the *k_i _*neighbours of node *I*. As *K_i_(k_i_-1)/2 *is the maximum number of such interactions, the clustering coefficient is a number between 0 and 1. The average clustering coefficient for a list of genes is obtained by averaging over the clustering coefficient of individual nodes. A network with a high clustering coefficient is characterized by highly connected sub-graphs.

#### Evaluation of one-step over (under)-connectivity between a protein with the protein list of interest

**Figure 3:**
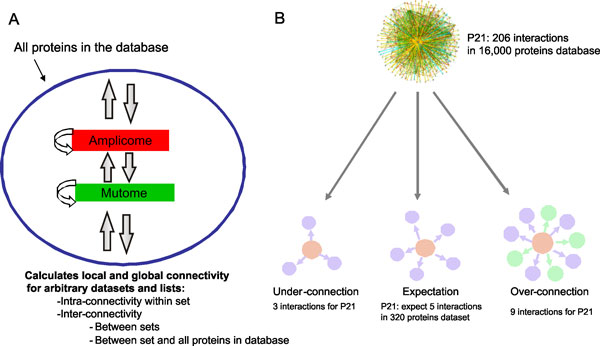
**Interactome analysis of proteomics datasets and gene lists**. A. The general schema of interactions inside the set, between the sets and between the set and "global interactome". B. The "over" and "under"-connectivity phenomenon. The hub (P21 protein from MetaCore database, marked pink) is expected to be linked with five other proteins in the hypothetical dataset of 320 genes (purple circles), but in reality is can be linked with nine genes (purple and green circles), or 3 genes (purple circles). In these cases, it will be considered as "over" connected or "under" connected.

It is widely accepted and shown in multiple studies that the most critical proteins in a given dataset (drug targets, disease-related proteins etc.) have more connections within the dataset than expected at random [18]. In MetaCore, we account for this observation in a statistical tool that evaluates relative connectivity of proteins of different functions. Relative connectivity is calculated for each protein as a ***ratio ***of an actual number of one-step protein-protein interactions (unique to the dataset) to the expected number of interactions, followed by summarizing interaction data for the whole dataset. Expected connectivity is dependent on the size of the dataset of interest, the total number of interactions the protein has and the total number of human proteins with at least one interaction. The *connectivity ratio *is, therefore, a quantitative measure of "functional relevance" of a protein for the dataset of interest. "Overconnected" transcription factors, ligands, receptors, kinases, phosphatases, proteases and metabolic enzymes are ranked based either on connectivity ratio or p-values (probability to come up with the observed connectivity ratio for a random dataset of the same size). The degree of over- and under-connectivity can be also evaluated by z-score, which signifies the difference between the obtained number of proteins and the expected average number of proteins being expressed in units of standard dispersion (Figure 3). Statistical significance is assigned by using the hypergeometric test. The null hypothesis is that a number of interactions of a node with the node set of interest do not exceed the number of interactions with a random set of nodes with the same size. P-value is calculated as follows:

$$\begin{array}{c} pVal\left(r,n,R,N\right)=\overset{\text{min}\left(n,R\right)}{\underset{i=max\left(r,\;R+n-N\right)}{\sum }}p\left(i,n,R,N\right),\;\text{where}\;\\ p\left(i,n,R,N\right)=\frac{\left(\begin{array}{c} R\\ r \end{array}\right)\left(\begin{array}{c} N-R\\ n-r \end{array}\right)}{\left(\begin{array}{c} N\\ n \end{array}\right)},\;\text{and} \end{array}$$

*N *is the number of proteins (protein-based network objects) in our global interactome extracted from MetaCore; *n *- number of proteins derived from the sets of genes of interest; *R *- the degree of a given protein in the global interactome database; r - the degree of a given protein within the set of interest.

The probability of observing under-connected proteins can be calculated by (*1 - p)*, where *p *is p-value for over-connection.

An example of overconnectivity analysis is demonstrated in Table 1. The list of differentially abundant proteins was obtained from serum of a mouse model of ovarian cancer [9] and subjected to "interactome by protein function" analysis in MetaCore. Given that this sample set is a profile of serum proteins, the prioritized interactome list, particularly the readily detectable list of ligands or receptors, was best suited for identifying potential biomarkers for early detection of ovarian cancer. The number of experimentally validated direct interactions is listed according to protein class (transcription factor, receptor, ligand, kinase, and protease) and in order of highest to lowest ratio in each category (Table 1).

**Table 1:** Interactome overconnectivity analysis of serum in ovarian cancer.

Object name	Actual	n	R	N	Expected	Ratio	p-value	z-score
Transcription factors								

Nkx5-1	2	79	9	19603	0.036	55.14	5.668E-04	10.33

NFIC	4	79	104	19603	0.420	9.54	8.277E-04	5.56

USF2	7	79	183	19603	0.738	9.49	9.061E-06	7.34

Receptors								

ITGB6	2	79	9	19603	0.036	55.14	5.668E-04	10.33

VLDLR	3	79	22	19603	0.089	33.84	9.180E-05	9.8

Glypican-1	2	79	16	19603	0.064	31.02	1.855E-03	7.64

Ligands								

LAMA2	2	79	7	19603	0.028	70.9	3.324E-04	11.77

CCL17	2	79	11	19603	0.044	45.12	8.614E-04	9.31

Thrombospondin 2	2	79	15	19603	0.060	33.09	1.627E-03	7.91

Kinases								

LRRK1	2	79	5	19603	0.020	99.26	1.591E-04	13.98

Proteases								

CTRL	1	79	1	19603	0.004	248.1	4.030E-03	15.72

Mcpt8	1	79	1	19603	0.004	248.1	4.030E-03	15.72

CRIM2	1	79	2	19603	0.008	124.1	8.044E-03	11.07

Actual = number of network objects in the activated dataset(s) which interact with the chosen object listed in object name column; n= number of network objects in the activated dataset( i.e. proteomics list); R=number of network objects in the complete database or background list which interact with the chosen object; N= total number of gene-based objects in the complete database or background list; Expected= mean of hypergeometric distribution (n·R/N); Ratio= connectivity ratio (Actual/Expected); z-score= (Actual-Expected)/s.d.(Expected), where s.d. - standard deviation of hypergeometric distribution, p-value= probability to have the given or higher (lower for negative z-score) value of Actual by chance under null hypothesis of no over- or under-connectivity.

**Figure 4:**
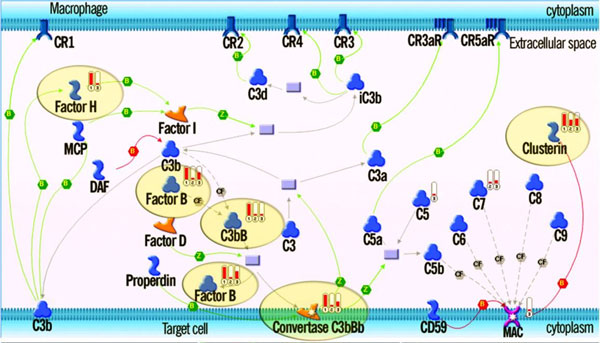
**Complement pathway activation in glaucomatous optic nerve astrocytes revealed by proteomics**. The relevant proteomic data (solid red indicator #1) and the differential gene expression data (indicators #2 and #3), mapped on the canonical pathway originally characterized in macrophages, show cross-verification of the complement pathway activation in glaucomatous ONHAs. Pathway steps confirmed by both data types are highlighted with ovals.

A one-step interactome analysis is also convenient for assessing the relationship between multiple datasets or data types, which poorly overlap at gene level. The topology based interactome tool helps to determine whether one data set is acting on another and in what direction. It was found that a large percentage of the 1188 genes with somatic mutations in breast cancer pooled from 11 exon-resequenced primary tumours were frequently upstream of a subset of the 1747 genes amplified in one of 30 breast cancer amplicons specifically in transcription regulation signalling. This finding suggested that mutated genes are mainly regulators, whereas gained genes are mostly regulated by physical one-step interactions. Furthermore, proteomics analyses are often paired with genomic or transcriptomics profiles to represent phenotypic changes across several levels of biology. For instance, Nikolskaya et al [19] reconstructed the molecular pathways of optic nerve head astrocytes in glaucoma by combining transcriptome interactome analysis with ontology enrichment and network generation (see Network analysis section) of well connected hubs defined by the proteomics analysis, resulting in the representation of the complement system by both data types (Table 2, Figure 4).

**Table 2:** Distribution of GO processes in glaucomatous optic nerve astrocytes revealed by proteomics

Top 10 GO processes (for 35 proteins)	p-value	Top 10 canonical maps (for 20 mapped proteins)	p-value
Complement activation. Alternative pathway	3.60E-06	CDC42 in cellular processes	1.10E-03

Response to heat	1.10E-05	Alternative complement pathway	1.70E-03

Nucleosome assembly	3.80E-05	Putative ubiquitin pathway	3.10E-03

Complement activations	4.10E-05	Role of ASK1 under oxidative stress	3.20E-03

Glycogen catabolism	5.40E-05	Role of IAP proteins in apoptosis	4.10E-03

DNA DSB repair via homologous recombination	1.00E-04	Glucocorticoid receptor signaling	5.10E-03

Chromosome organization	1.40E-04	Role of Akt in hypoxia	7.80E-03

Carbohydrate metabolism	1.50E-04	Parkin disorder under Parkinson's disease	8.10E-03

Innate immune response	2.20E-04	Role of Parkin in the ubiquitin-proteosomal family	8.10E-03

Small GTPase mediated signal transduction	3.10E-04	Urea cycle	3.40E-02

#### "Hidden nodes" topology analysis: algorithm for node prioritization and reconstructing significant pathway modules

Analysis of relative connectivity described above is limited to one-step interactions which define the local interaction "neighbourhood" for each protein. This topology evaluation procedure can be extended to encompass several steps of signalling mechanisms, eventually covering all human proteins with respect to the connectivity (expressed as p-values) within the proteomics dataset. We call this approach a "hidden nodes" analysis, as most of the highly ranked proteins revealed by this method miss from original proteomics data [20]. As in the case of one-step interactome, the scoring is based on the role the protein nodes play in connectivity among genes or proteins of interest relative to their role in the *global network*. The method is neutral with respect to the node's degree or centrality, i.e., the role of nodes with a high degree of physical connections (such as p53 or NF-kB) is normalized on the entire interactome. The scores for truly significant nodes are enhanced, while the scores of those that appear in the networks by chance are reduced. The output of "hidden nodes" analysis is a set of prioritized proteins divided onto functions along with their possible regulatory effects on the proteins from a proteomics profile. A user receives a series of scored and testable hypotheses associating individual components of the identified molecular network(s) with the phenotype of interest.

### Topological scoring of nodes

**Figure 5:**
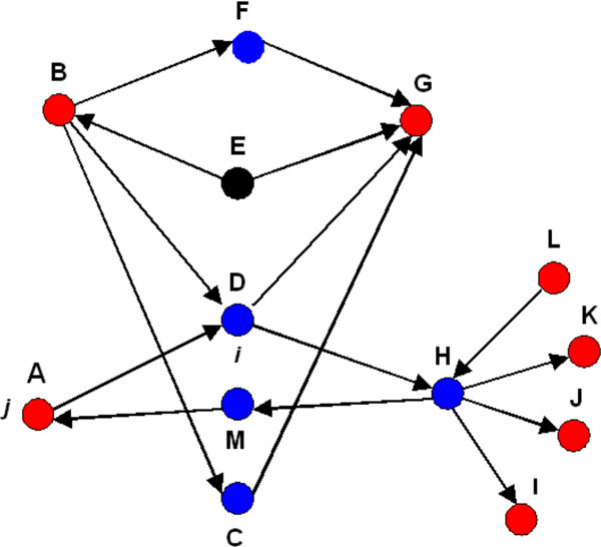
**A diagram for hidden nodes algorithm**. A set of experimentally derived nodes ***K ***is colored red. We connect them by shortest path network ***S ***(blue nodes). The rest of the global network is represented by black nodes.

The topological scoring algorithm starts with a set of experimentally identified genes or proteins as the seed nodes (K). K is a subset (a number of nodes) of a global network of size N. The first step is the construction of a directed shortest path network connecting each node in K to other nodes in K, traversing via other nodes in the global network. If there are multiple shortest paths of equal length between two nodes within K, then all of the nodes from the multiple paths are included in the shortest path network for that pair, S, which is a subset of N and contains nodes in addition to K. Some nodes from K may become "internal" in S, that is, they are lying on the shortest paths, while the rest are either "source" or "target" terminals of the shortest paths (Figure 5). All nodes in S that are not in K are by definition "internal" nodes. For future reference, we call S a condition-specific shortest path network (CSSPN). The shortest path network is built by a modified version of the standard breadth-first search described elsewhere [12]. Let us consider node *i *∈ S, an internal node, and j ∈ K, one of the nodes of the experimental set. In addition to S, we calculate the shortest paths between *j *and every other node (except i) in the global network, wherever such shortest paths exist (up to N-2 pairs). Then we count how many of these node pairs have node *i *present in at least one of the shortest paths; this number is N*_ij _*≤ N-2. On the other hand, we count how many times node *i *occurs in at least one shortest path of node pairs when connecting *j *to all other nodes in K. This number is K*_ij _*≤ K_j _≤ K-1 (we assume node i is not differentially expressed; otherwise K*_j _*≤ K-2). Note that we count node *i *only once for every pair from K, even though it may be part of multiple linear shortest paths connecting the same pair. Under the "null" hypothesis, node *i *has no special role in connecting node *j *to the rest of differentially expressed genes in K. Thus, the probability of finding *i *in the shortest paths connecting K*_ij _*or a larger number of node pairs originating or terminating at node *j *follows a cumulative hypergeometric distribution. This problem can be recast as selection without replacement. N*_ij _*node pairs containing *i *as an internal node in the shortest paths connecting *j *to all other nodes in the global network can be considered as a set of "marked" node pairs. On the other hand, a set of K-1 pairs consisting of the node *j *and the rest of K- 1 experimentally derived nodes represent a "selection". If node *i *has no special role for connecting *j *to the rest of the nodes in K, then the number of marked shortest path networks in the selection should follow the hypergeometric distribution where p*_ij_*(K*_ij_*) is the probability of finding node *i *in the shortest paths connecting K*_ij _*number of node pairs in the differentially expressed set among those originating or terminating at node *j*.

$$p_{ij}\left(K_{ij}\right)=\frac{\left(\begin{array}{c} N_{ij}\\ K_{ij} \end{array}\right)\left(\begin{array}{c} N-N_{ij}-2\\ K-K_{ij}-1 \end{array}\right)}{\left(\begin{array}{c} N-2\\ K-1 \end{array}\right)}$$

The null hypothesis of scoring test is that node *i *has no special role in connecting *j *with other nodes of interest via shortest paths. The p-value of the test is calculated as cumulative probability of observing 'K_ij_' or more paths by chance under the null hypothesis. We repeated this procedure for all nodes in K, calculating up to K p-values for each node i in the network of shortest paths connecting differentially expressed genes. Each of these p-values shows relevance of node *i *to individual members of the set K. As we want to identify the nodes that are statistically significant to at least one or more members of the experimental set, we define the "topological significance" score associated with node *i *as the minimum of the p_ij _values. We note that our method, unlike betweenness centrality, does not count the actual number of shortest paths between the pairs of nodes, but rather it counts the number of instances a node is part of the shortest path network between the node pairs. More importantly, our technique considers fractions of differentially and non-differentially expressed genes connected by shortest paths containing the node that is being evaluated. In this context it is not concerned with the paths bypassing the node of interest. In contrast, the betweenness centrality measure is based on relative numbers of shortest paths going via the node of interest and those bypassing it.

For example, in Figure 5, the size of the global network *N *= 13, *K *= 7, and *S *= 5. The number of possible shortest path networks between node *B *and each of the other nodes in the global network, which can contain D, are 11 (*N*-2). The number of such networks which contain node *D *is 7 (*NBD *= 7). On the other hand, the number of shortest path networks containing *D*, among those connecting only nodes from the set *K*, is 5 (*KBD *= 5). The significance (p-value) for node *D *with respect to node *B *and set *K *can be calculated as *pBD *= *p*(*N*-2, *NBD*, *K*-1, *KBD*). Similarly, we can calculate the other p-values for *D *with respect to *A, G*, *K*, *J*, *I*, and *L*, and then pick the smallest value and assign it as the significance of node *D *in the sub-network defined by the nodes of interest (red nodes). The nodes can be classified as internal (*F*, *D*, *C*, *H*, and *M*), source (*A*, *B*, and *L*) and target (*A*, *G*, *K*, *J*, and *I*) nodes.

The hidden nodes approach is another example that supports and integrates functionality of several data types. Vellaichamy et al recently [21] reconstructed system-wide molecular events following stimulation of LNCaP prostate cancer cells with synthetic androgen to identify potential mechanisms of androgen-independent progression of prostate cancer using concurrent measurements of gene expression and protein levels from microarrays and iTRAQ proteomics techniques, respectively. The list of up-regulated genes and proteins were submitted to the scoring procedure separately, resulting in two sets of topologically significant regulatory proteins. A total 962 topologically significant proteins from gene expression data and 577 topologically significant proteins from proteomic data (FDR<5%) were identified. Of these two sets of topological significant nodes, 301 were common, (or 52% of the smaller set) demonstrating a more than 2-fold increase in overlap between data types (17% overlap between lists of up-regulated genes and up-regulated proteins) (Table 3).

**Table 3:** The highest scored topologically significant proteins from gene expression and proteomics data for androgen-stimulated LNCaP prostate cancer cells

Symbol	Entrez Gene ID	Description	p value
AAG11	2274	aging -associated gene 11	2.24E-19

CLS	6197	ribosomal protein S6 kinase, 90kDa, polypeptide 3	1.51E-18

CTNNB	1499	catenin (cadherin -associated protein), beta 1, 88kDa	5.45E-17

CCND2	894	cyclin D2	3.74E-16

FLJ21396	23132	RAD54 -like 2	5.26E-16

AIS	367	androgen receptor	1.36E-15

DAB2	1601	mitogen -responsive phosphoprotein	1.55E-15

ABP -280	2316	filamin A, alpha	3.89E-15

ACH	2261	fibroblast growth factor receptor 3	1.2E-14

RAD9	5883	RAD9 homolog	1.31E-14

			

**Symbol**	**Entrez Gene ID**	**Description**	**p value**

MYC	4609	myc proto-oncogene protein	2.15E-08

HIRS-1	3667	insulin receptor substrate 1	1.85E-07

BCL2	596	B-cell CLL/lymphoma 2	4.21E-07

INSRR	3645	IR-related receptor	4.34E-07

MGC26306	9414	tight junction protein 2 (zona occludens 2)	4.38E-07

GCCR	2908	glucocorticoid receptor	5.98E-07

IRF-1	3659	interferon regulatory factor-1	1.01E-06

SREBF1	6720	sterol regulatory element binding transcription factor 1	1.06E-06

FLJ12859	23528	ZNP-99 transcription factor	1.1E-06

ETV3	2117	ets variant gene 3, ETS family transcriptional repressor	1.12E-06

The top table contains Entrez Gene IDs, gene symbol, gene names and topological scoring test p-values for top 10 topologically significant proteins identified for overexpressed gene list. The bottom table contains the same information for 10 top topologically significant proteins identified for overexpressed protein list.

**Figure 6:**
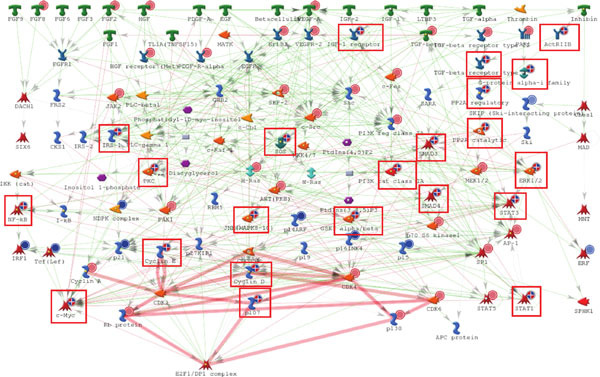
**"Growth factor regulation of the G1-S transition in cell cycle" network in LNCaP prostate cancer cells**. The red dots indicate proteins identified as topologically significant using the gene expression profile. Blue dots indicate proteins identified as topologically significant using the proteomics profile. Red boxes-proteins identified as topologically significant from both sets of data.

The resulting topologically significant proteins from a hidden nodes analysis can be combined with ontology enrichment to determine the differences of functionality between data types (genes vs. proteins) or as a combination. In general, the top pathways represented as a consequence of gene or protein expression differed but complemented each other at different levels of connectivity. For instance, in the case of the LNCaP prostate cancer cell analysis, the "Growth factor regulation of cell cycle" network (Figure 6) is highly enriched in proteins that are topologically significant for both gene expression and proteomic data while the androgen signalling network was more represented by the proteins that are topologically significant for gene expression. Close examination of this process reveals that topologically significant proteins are present on all levels of signalling hierarchy, including several growth factors, receptors, signalling kinases, transcription factors and cyclin kinases. The overall impact of these findings emphasizes the need to identify interconnected network modules containing many alternative routes and various levels of biology for the precise formulation of combination therapy to effectively fight the tumour growth.

### Network analysis of proteomics data

Network analysis is another method assessing connectivity within a given dataset such as a proteomics list. In MetaCore, networks are generated as a combination of binary single step interactions (edges or links), which connect network objects (nodes). In generating networks, a user's dataset (for instance, a proteomics profile) is considered as a list of root nodes (any protein, gene or metabolic ID) and MetaBase is used as the source of interactions as edges between them. As the root node lists are different, the generated networks are unique for the uploaded datasets and chosen conditions, which makes networks a quite flexible and precise research method. The same dataset (list of root IDs) can be connected by interactions in different ways, depending on a chosen network parameters and filters. The MetaCore's network toolbox features several network algorithms (each with a specific statistical method) and filters enabling generation of networks specific for cellular processes, species, orthologs, cellular processes, expression in human tissues, mechanisms of interactions and effects. The end nodes on the networks have only one edge; the internal nodes may have anywhere from two to several hundred edges.

#### Network generation algorithms

**Figure 7:**
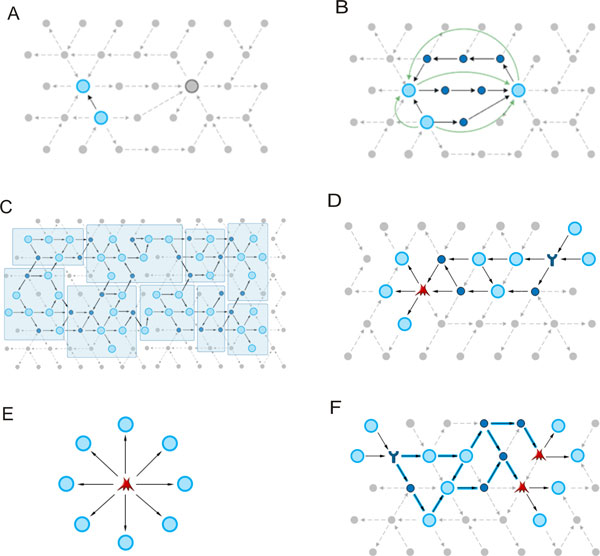
**Diagrams of network algorithms in MetaCore**. (A) Direct interactions algorithm; (B) Shortest path algorithm; (C) Analyze network; (D) Analyze network (Transcription Factors); (E) Transcription regulation; (F) Analyze network (Receptors).

On average, human proteins have over 20 interactions, with thousands of interactions for some highly connected proteins and complexes such as p53, NF-KB, AP1, PIP3K. With 24,000 human proteins with at least one interaction and over 500,000 experimentally confirmed interactions in total (MetaBase, Thomson Reuters), the complexity of interaction space is overwhelming, with billions of possible multi-step connectivity combinations even for a relatively small set of proteins in proteomics profiles. In order to select the most probable combinations of interactions activated in a given experiment, one can apply certain rules the directed interactions can be connected by. These rules are known as network generation algorithms. In MetaCore, there are seven basic network algorithms and two additional versions (Figure 7).

##### Direct interactions (DI) algorithm

**Figure 8:**
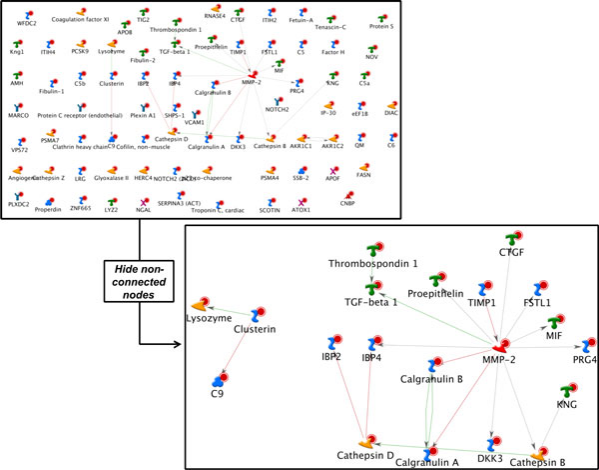
**Direct interaction network of a plasma proteome from a mouse ovarian cancer model**. 58 proteins were used to determine the direct relationship within the input list. A total 19 proteins were found to be directly interacting within 2 separate clusters. Directional edges are marked by colored arrows (green = activation, red = inhibition).

This is the most stringent algorithm (Figure 7), which allows visualization of only the edges connecting root nodes. The density of a direct interaction network is a function of the average number of edges per node in the source interaction database. With an input list of a substantial size (over 200 nodes), the assembly is typically presented as one large network along with several smaller clusters and a number of non-connected nodes. It is important to note two critical aspects of the DI algorithm 1) this algorithm will consider only the provided root list in network generation and 2) this algorithm may miss interactions which are essential for the network topology and biologically relevant, but not deducible from the original input list of objects as they are not directly connected. For example, when the input nodes represent differential expression data, the DI algorithm may miss many regulatory interactions at the level of protein activity, such as those that are relevant to proteomics including phosphorylation/dephoshorylation interactions, which are usually not associated with elevated transcription levels (as represented by differential expression data). For instance, let us consider the proteomics list of 58 proteins derived from the plasma of the ovarian cancer mouse model example above [15] (Figure 2). The DI network of this set of proteins suggests that a large percentage is NOT directly connected and upon closer examination, two centralized hubs exist (Figure 8). From this, we can prioritize MMP-2 and Clusterin as potential proteins for further biomarker development, based on connectivity.

##### Expand by One Interaction

This algorithm builds one-step sub-networks around any object from the list. The algorithm helps to identify direct upstream and direct downstream effectors, then finds "islands" of nodes from the user's list connected by no more than two bridging objects.

##### Shortest path (SP) algorithm

This is a less stringent algorithm than the DI as it allows for the addition of non-root nodes to the networks (objects from the database, not originally in the original user file). The SP algorithm works as follows: when there are two lists of nodes, one for the initial nodes, and another for end nodes, the lists are considered as almost always identical and corresponded to the input list. For every node from the initial list, the set of shortest paths (chains of consecutive directed interactions) to every other node from the 'end nodes list' is established. For every pair of nodes, all of the minimal paths are built and depicted as an inter-connected network of pathways. The number of steps defined by user options can limit the length of paths.

##### Auto-expand (AE)

AE algorithm creates sub-networks around every object from the uploaded list. The expansion halts when the sub-networks intersect (whether it is 1-step or more). The objects that do not contribute to connecting sub-networks are automatically removed.

##### Analyze Network (AN)

This algorithm starts with building a super network by applying a simplified version of the "Auto Expand" algorithm to the initial list of objects. The network, which is never visualized as a whole, *connects all objects from the input list with all other objects in the input list*. Naturally, this process results in a super-connected large network, and is therefore "divided" into smaller fragments of a user-chosen size, from 2 to 100 nodes. The division process is conducted in a cyclical manner, i.e. fragments are created sequentially one by one where edges used in a fragment are never reused in subsequent fragments. Nodes may be reutilized, with different edges leading to them in different fragments. The end result of the AN algorithm is a list of multiple overlapping networks (usually ~30), which can be prioritized based on five parameters: the number of nodes from the input list among all nodes on the network, the number of canonical pathways on the network, and three statistical parameters: p-value, z-score and g-score (see network statistics below).

The resulting networks are significantly different from those generated by the DI algorithm and address different "connectivity' questions. In the AN algorithm, a user is looking for a more explorative network of interactions based on the logical progression of cellular signalling, using the database as a resource. Therefore, each division may represent a different collective function. For example, using the same input list defined in the DI example, the AN table shown in Table 4 suggests the first two sub-networks represent regulation of biological quality yet, each contains individual representations of morphogenesis or inflammation (network 1 and 2, respectively).

**Table 4:** Analyze network' algorithm results for a plasma proteome from a mouse ovarian cancer model

No	Network	GO Processes	Total nodes	Seed nodes	Pathways	p-Value	zScore	gScore
1	Factor H, APOF, Cathepsin Z, TIG2, SSB-2	positive regulation of cell adhesion (15.6%; 1.020e-08), regulation of biological quality (44.4%; 1.296e-08), response to inorganic substance (22.2%; 7.552e-08), wound healing (17.8%; 2.420e-07), anatomical structure morphogenesis (35.6%; 3.159e-07)	50	14	2	1.270E-29	54.15	56.65

2	Fetuin-A, IBP4, Thrombospondin 1, KNG, SERPINA3 (ACT)	response to wounding (39.6%; 8.503e-15), regulation of biological quality (54.2%; 2.556e-13), response to stimulus (72.9%; 3.053e-12), inflammatory response (29.2%; 3.365e-12), response to stress (52.1%; 1.913e-11)	50	14	0	2.470E-29	53.05	53.05

3	Calgranulin A, Calgranulin B, VCAM1, Cathepsin D, NOTCH2	positive regulation of cellular process (67.4%; 1.338e-15), response to stress (62.8%; 5.497e-15), positive regulation of biological process (67.4%; 1.566e-14), multicellular organismal process (83.7%; 5.605e-14), developmental process (74.4%; 9.693e-14)	50	13	0	8.090E-27	49.24	49.24

4	Thrombospondin 1, CNBP, AKR1C1, Coagulation factor XI, NOV	regulation of biological quality (58.8%; 1.894e-11), multicellular organismal process (79.4%; 8.590e-10), regulation of multicellular organismal process (44.1%; 1.560e-08), response to stress (50.0%; 7.234e-08), positive regulation of cellular component organization (23.5%; 1.432e-07)	50	11	2	3.690E-22	42.49	44.99

An excerpt of AN network list (from MetaCore) constructed from the list of 58 proteins derived from a plasma proteome of a mouse ovarian cancer model. For a complete description of each column refer to network statistics please see "3.2.2. Prioritization of sub-networks".

**Figure 9:**
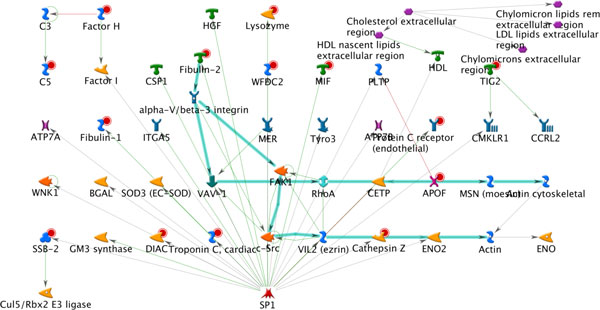
**The top scoring network of AN algorithm applied to a plasma proteome from a mouse ovarian cancer model**. Directional edges are marked by colored arrows (green = activation, red = inhibition) and root/input nodes are marked with red circles. Teal blue arrows delineate presence of interactions that represent canonical, well-established mechanisms

A closer look at the first network illuminates a different set of connections, compared to the DI MMP-2 -rich network (Figure 9). As a consequence, several different and distinct observations can be made: 1) this network was determined as the most significant based on g-score (which considers representation of well established signalling mechanisms); 2) several proteins can be prioritized based connectivity to a canonical mechanism marked by the teal blue lines (as opposed to hub connectivity determined in the DI example) including Fibulin-2 and Cathepsin-Z; and 3) the series of interactions, including the marked canonical mechanism are influenced by the presence of sp-1, providing yet another level of prioritization for transcriptional regulation purposes. Such observations greatly contribute to subsequent stages of discovery or drug development.

##### Analyze network (Transcription Factors - TFs) and Analyze network (Receptors)

Both algorithms start with creating two lists of objects expanded from the initial list: the list of transcription factors and the list of receptors. Next, the algorithm calculates the shortest paths from the receptors to TFs. Then, the shortest paths are prioritized in a similar way. The first algorithm, AN (TFs), connects every TF with the closest receptor by all shortest paths and delivers one specific network per TF in the list. Similarly, the second algorithm AN (R ) delivers a network consisting of all the shortest paths from a receptor in the list to the closest TF; one network per receptor. Since all the edges, and therefore, paths are directional, the resulted networks are not reciprocal.

Every network built by an AN algorithm may be optionally enriched with the receptor's ligands and the TF's targets. The networks may be grouped, and merged within every group. Namely, if we are building one network for every transcription factor, then all such networks with the same receptors are grouped and merged within each group.

##### Transcription regulation (TR)

This algorithm starts with a small sub-network that consists of the initial list of objects plus all the "immediate transcription factors" for those initial objects, i.e. the objects that are linked to at least one of the initial objects by an edge of the "transcription regulation" type. Then, a separate network is built around every such transcription factor, using the AE algorithm with "upstream" option and limiting to the objects from the initial list. Then the transcription factor's targets from the initial list are added to network. The algorithm delivers a list of networks, one per transcription factor.

##### Model canonical pathways

MetaBase contains a library of pre-reconstructed "canonical" signaling pathways. Each pathway represents a linear chain of signal transduction interactions thoroughly described in the literature; 17 steps on average. Each object n-1 in the chain is connected with object n by one outgoing interaction. The pathway typically starts with a ligand and its corresponding receptor and ends with a transcription factor and its target gene. The "model canonical pathways" algorithm attempts to reconstruct the networks, which are composed exclusively of canonical pathways and enriched with objects from the input list. The main steps are as following:

- Determine all objects from the input list that are involved in at least one canonical pathway;

- For each selected object, retrieve all canonical pathways containing this object and merge them into a separate network (each network is built around a single root object from the input list;

- Optionally, add objects from the input list that is regulated by transcriptional factor already present in network;

- Optionally, add objects from the input list that binds to the receptor already present in network;

- Rank networks by the number of input list objects they contain. Networks with equal number of such objects are ranked by size (the less total number of objects, the higher rank).

#### Prioritization of sub-networks

Application of network ranking statistics is important for the analysis of large high throughput experimental datasets, such as expression profiles or proteomics datasets, which may include hundreds to thousands of proteins and complexes. The networks can be ranked using the z-scores calculated from the hypergeometric distribution. These scores reflect the relative enrichment of reconstructed networks by the genes/proteins from the list of interest. Let us consider a general set size of N with R marked objects/events (for example, the nodes with expression data). The probability of a random sub-set of size of n which includes r marked events/objects follows the hypergeometric distribution and is calculated as

$$p\left(r,n,R,N\right)=\frac{\left(\begin{array}{c} R\\ r \end{array}\right)\left(\begin{array}{c} N-R\\ n-r \end{array}\right)}{\left(\begin{array}{c} N\\ n \end{array}\right)}$$

The mean of this distribution is equal to the following:

$$\mu =\overset{n}{\underset{r=0}{\sum }}r\cdot P\left(r,n,R,N\right)=\frac{n\cdot R}{N}=n\cdot q,$$

where q=R/N defines the ratio of marked objects. The dispersion of this distribution is described as follows.

$$\sigma^{2}=\overset{n}{\underset{r=0}{\sum }}r^{2}\cdot P\left(r,n,R,N\right)-\mu^{2}=\frac{n\cdot R\left(N-n\right)\cdot \left(N-R\right)}{N^{2}\cdot \left(N-1\right)}=n\cdot q\cdot \left(1-q\right)\cdot \left(1-\frac{n-1}{N-1}\right)$$

It is essential that these equations are invariant in terms of exchange of n for R. This means that the "subset" and "marked" are the equivalent and symmetrical sets.

We use the following z-score for prioritization of node-specific SP sub-networks.

$$z-score=\frac{r-n\frac{R}{N}}{\sqrt{n\left(\frac{R}{N}\right)\left(1-\frac{R}{N}\right)\left(1-\frac{n-1}{N-1}\right)}}=\frac{r-\mu }{\sigma }$$

Where:

*N *is the total number of nodes after filtration;

*R *is the number of nodes in the input list or the nodes associated with experimental data;

*n *is the number of the nodes in the network;

*r *is the number of the network's nodes associated with experimental data or included in the input list;

*µ *and σ are respectively, the mean and dispersion of the hypergeometric distribution described above.

## Conclusions

Here, we described the basics of analysis of proteomics data by functional methods. Essentially, all functional analysis is divided to "protein-based" and "interaction-based" methods. The former consists of relative enrichment of a list of proteins (proteomics profile) with the terms of functional ontologies, such as pathways, cellular processes and disease biomarkers. This is a low resolution, descriptive analysis, helpful for the first look at the data and functional filtering of proteomics profiles. This type of analysis is useful for basic biology applications and relatively large protein sets; preferably over 200 proteins. Enrichment profiles can also be applied quantitatively, with one or several top scoring ontology terms representing a functional descriptor. Pathway descriptors can be applied for clinical sample clustering or prediction of disease prognosis or toxic effects (reviewed at [16]).

The interaction-based methods are based on the assumption that it is the set of physical interactions that defines the protein functionality in a living cell. Therefore, evaluation of the local "interactome" for each protein from proteomics datasets could be used as a flexible ad powerful research tool. High resolution interactome analysis is well applicable for drug target identification and biomarker discovery. Although technically there is no size limit, Interactome and network tools work best on relatively small protein sets and particularly useful when the sample size is too small for statistical analysis of sufficient power. Deducing companion biomarkers for drug response in clinical trials is a typical application. In most clinical study settings, drug sensitivity and resistance biomarkers have to be identified from a limited number of Phase I or II samples (or pre-clinical *in vitro *assays) and validated in much larger and more expensive Phase III studies. A small sample size often makes statistical tools for "gene signature" calculations irrelevant, and researchers have to rely on interactome-based methods, such as "causal reasoning" [22].

## Competing interests

The authors declare that they have no competing interests.

## Authors' contributions

MB - main contribution to writing text, algorithm development; AI - statistical analysis; LJ - major contributor in ovarian cancer study; wrote about ovarian cancer example; TN - major contributor in glaucoma study; wrote about glaucoma example;

YN - project leader; wrote and edited most of text.
